# Regulatory landscape of accelerated approval pathways for medical devices in the United States and the European Union

**DOI:** 10.3389/fmedt.2025.1586070

**Published:** 2025-05-15

**Authors:** Tanvi Gupte, Tushar Nitave, Jogarao Gobburu

**Affiliations:** ^1^ Center for Translational Medicine, University of Maryland School of Pharmacy, Baltimore, MD, United States; ^2^ Department of Translational Medicine, Vivpro.ai, Jersey City, NJ, United States

**Keywords:** breakthrough devices, accelerated approval, medical devices, harmonization, Medical Device Regulation (MDR), Food and Drug Administration (FDA), Health Technology Assessment (HTA)

## Abstract

The landscape of medical device regulation is rapidly evolving, driven by innovations and the need to bring these technologies to patients more efficiently. This review provides a comprehensive analysis of the accelerated access pathways for medical devices in the United States (US) and the European Union (EU), focusing on the Breakthrough Devices Program (BDP) in the US and the evolving regulatory framework within the EU. Analysis of Food and Drug Administration (FDA) data reveals that from 2015 to 2024, only 12.3% of the 1,041 BDP-designated devices received marketing authorization, with mean decision times of 152, 262, and 230 days for 510(k), *de novo*, and PMA pathways respectively—significantly faster than standard approvals for *de novo* (338 days) and PMA (399 days). In the EU, where no specific accelerated pathway exists, the recently implemented Medical Device Regulation and Health Technology Assessment Regulation aim to harmonize approval processes, with joint clinical assessments beginning in 2026. The analysis explores the interplay between regulatory approval, funding mechanisms, and coverage policies that collectively determine the accessibility of medical devices. The unique challenges associated with emerging technologies and the implementation of accelerated pathways are also discussed. We recommend global regulatory convergence through harmonized standards, mutual recognition agreements, and unified post-market surveillance systems to balance innovation with patient safety.

## 1 Introduction

The medical devices and device-led combination products sector is characterized by continuous advancements and improvement of existing technologies fuelled by research and development (R&D) activities and strong collaboration between manufacturers, healthcare professionals, health insurance and reimbursement authorities, and end-users. This is a fast-evolving sector with innovations ranging from implants and wearables to complex combination products that integrate drugs, devices, and biologics. These innovations aim to address medical needs, enhance patient outcomes, and improve the efficiency of patient care processes.

**Figure 1:**
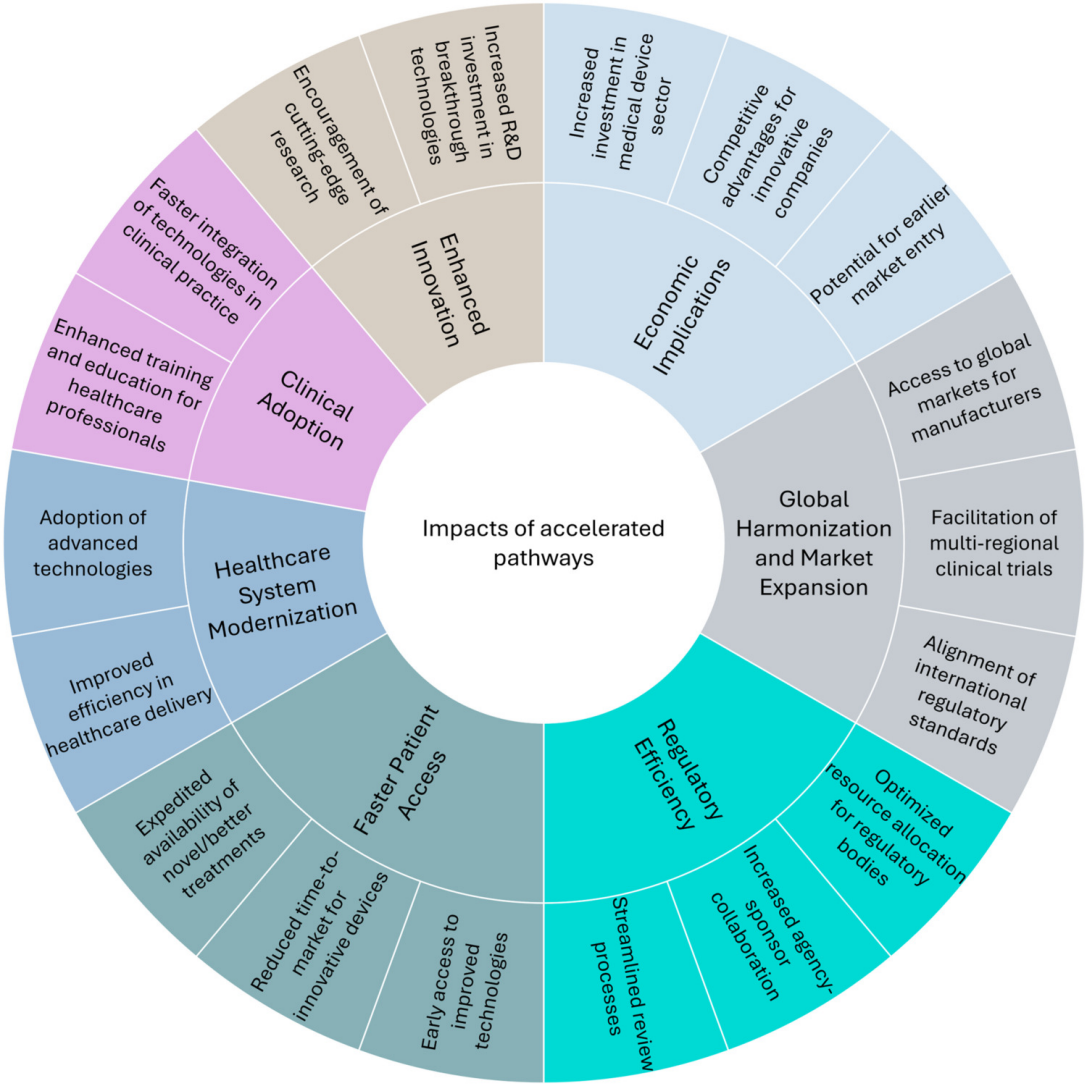
Impacts of accelerated pathways on global healthcare.

Regulatory agencies around the world have recognized the need to expedite public access to innovative, lifesaving, and/or more effective medical technology. Regulatory frameworks and accelerated pathways have evolved to support the clinical integration of these emerging technologies. These pathways aim to streamline regulatory processes while ensuring the safety, efficacy, and quality of medical devices. Such pathways not only enable a faster market entry but also encourage innovation, which is especially necessary for serious health conditions and rare diseases. The U.S. Food and Drug Administration (FDA), for instance, introduced the Breakthrough Devices Program (BDP) to fast-track the review of medical devices that provide more effective treatments or diagnosis of life-threatening or irreversibly debilitating diseases (1). The European Union (EU) has recently introduced the Health Technology Assessment Regulation (HTAR) for expedited, evidence-based decision-making and better access to innovative treatments (2). Similar expedited pathway initiatives have been developed in other countries to address the increasing demand for innovative solutions in healthcare. As the sector continues to advance, expedited pathways are expected to create widespread impacts across healthcare systems, benefiting patients, regulators, and the industry alike. Figure 1 illustrates the key impact areas where accelerated pathways are reshaping healthcare on a global scale. These pathways create widespread effects across clinical adoption, healthcare system modernization, regulatory efficiency, economic implications, and global harmonization. While the figure highlights the positive impacts, such as faster regulatory authorization, it is important to acknowledge the downstream challenges these pathways create. These include not only potential safety concerns from limited pre-market evidence but also considerable obstacles for reimbursement authorities who must evaluate technologies and assess cost-effectiveness with limited effectiveness data. This evidence uncertainty frequently results in a disconnect between regulatory approval and patient access, as coverage decisions may be delayed or restricted despite regulatory approval until real-world performance data becomes available. The effectiveness and impact of these expedited pathways will likely be subject to ongoing evaluation and refinement.

It is important to note that focusing solely on regulatory requirements may lead to oversight of broader stakeholder expectations, potentially affecting coverage determinations and clinical adoption. A comprehensive approach to quality healthcare requires:

• Regulatory pathway: to streamline the approval process while ensuring safety and efficacy.
• Coverage mechanisms: to determine whether a medical technology will be reimbursed, and to what extent. Also, to ensure timely patient access and affordability through health insurers and national health systems.

For instance, the Centers for Medicare & Medicaid Services (CMS) makes critical coverage decisions for FDA-approved devices in the United States. Failing to include sufficient Medicare beneficiaries in clinical trials could result in challenges in obtaining a positive National Coverage Determination (NCD), which is required for Medicare payment (3). Similarly, in the European Union, the HTAR plays a key role in evaluating the clinical and cost-effectiveness of new technologies. To ensure smooth transitions from regulatory approval to coverage and reimbursement, medical technology developers should consider the requirements of both regulatory bodies and funding agencies throughout the development and evidence-generation process. This approach ultimately facilitates faster patient access to innovative medical technologies.

This review examines the evolving landscape of accelerated regulatory pathways for medical devices in the United States and the European Union. We analyze key initiatives designed to expedite market access for innovative devices that address critical unmet medical needs while maintaining essential safety and efficacy standards. Our analysis begins with a comprehensive look at the FDA's BDP, exploring its impact on approval timelines and associated challenges. This is followed by the description of the EU's regulatory framework, including the Medical Device Regulation (MDR) and recent updates about the HTAR, which aim to harmonize approval processes across member states.

This review is particularly relevant as healthcare systems worldwide strive to balance innovation with patient safety. Understanding these accelerated pathways is crucial for medical device manufacturers navigating complex regulatory environments and for policymakers seeking to optimize regulatory processes. By critically examining the strengths and limitations of these programs, including their funding mechanisms and coverage implications, we aim to contribute meaningful insights to the ongoing dialogue on regulatory efficiency in an era of rapid technological advancement in healthcare.

## 2 Methodology

The primary objective of this review is to provide a comprehensive analysis of medical device accelerated approval pathways in the US and EU.

The source selection process focused on:

### 2.1 Source types

• Primary sources:
  ○ Official regulatory documents from FDA and EU regulatory bodies
  ○ Federal registry publications
  ○ Regulatory guidance documents
• Secondary sources:
  ○ Peer-reviewed academic literature
  ○ Policy reports from reputable medical technology organizations

### 2.2 Selection criteria

Sources were selected based on the following key criteria:

• Issued between 2015 and 2025
• Directly relevant to medical device regulatory pathways
• Focused on accelerated approval mechanisms
• Authored by recognized regulatory experts or official government agencies

## 3 Accelerated approval in the US - Breakthrough Devices Program

The Breakthrough Devices Program (BDP) is an initiative by the U.S. Food and Drug Administration (FDA) to accelerate the development and review of innovative medical devices in the US. It is a voluntary program introduced in 2015 and formalized under the 21st Century Cures Act of 2016, replacing the Expedited Access Pathway (EAP) (1). Devices granted designation under EAP were transitioned to the BDP since the goals and criteria for the two programs are similar.

BDP intends to speed up the development, assessment, and review of medical devices while still meeting the FDA's requirements for safety and effectiveness. It applies to devices and combination products that are subject to review under a premarket approval (PMA), 510(k) clearance, or *de novo* classification, including devices regulated by Center for Biologics Evaluation and Research (CBER). To qualify for the BDP, a device must meet two primary criteria:

1. It must provide for more effective treatment or diagnosis of life-threatening or irreversibly debilitating human diseases or conditions.
2. It must satisfy at least one of four secondary criteria: represent breakthrough technology, offer significant advantages over existing alternatives, address an unmet medical need, or its availability must be in the best interest of the patient (1).

To apply for BDP designation, the companies are required to submit a request using the Q-submission program (4). The FDA then evaluates the request and provides feedback. The BDP consists of a designation phase, where a device is granted breakthrough status, and a development phase, which focuses on expedited development and prioritized regulatory review. The program has evolved to address emerging healthcare challenges and priorities.

In September 2023, the FDA updated its guidance to clarify how the program applies to devices that may address health inequities, aligning with the Center for Devices and Radiological Health's (CDRH) priority to advance health equity (1). This update emphasizes the consideration of technologies and device features that may help address health and healthcare disparities. Moreover, the program now considers devices designed to address pathophysiological or clinical characteristics associated with certain populations, devices tailored for rare conditions with limited treatment options, and those offering improved accessibility for diverse populations or settings. The BDP has also been expanded to include certain non-addictive medical products for treating pain or addiction, in line with the FDA's obligations under the SUPPORT Act (1). This expansion reflects the program's approach to critical public health needs.

From 2015 to 2024, the FDA has granted breakthrough designation to 1,041 devices (1), including devices under the precursor EAP (*n* = 26) (5), demonstrating the program's significant impact on accelerating innovation in the medical device industry. Breakthrough device designation precedes marketing authorization and may precede human clinical studies (5). The FDA does not publicly disclose the list of devices granted BDP designation; instead, it is up to the manufacturers' discretion to share this information. However, BDP devices with marketing authorization are accessible. As of September 2024, only 12.3% of 1,041 BDP-designated devices have received marketing authorization (*n* = 128) (1). This can be attributed to the inherent rigorous evidence requirements for safety and effectiveness. Despite the priority review and additional FDA feedback provided by the BDP designation, devices may face delays or rejection if they cannot meet these requirements. This can be particularly challenging for innovative devices that lack extensive pre-market data.

**Figure 2:**
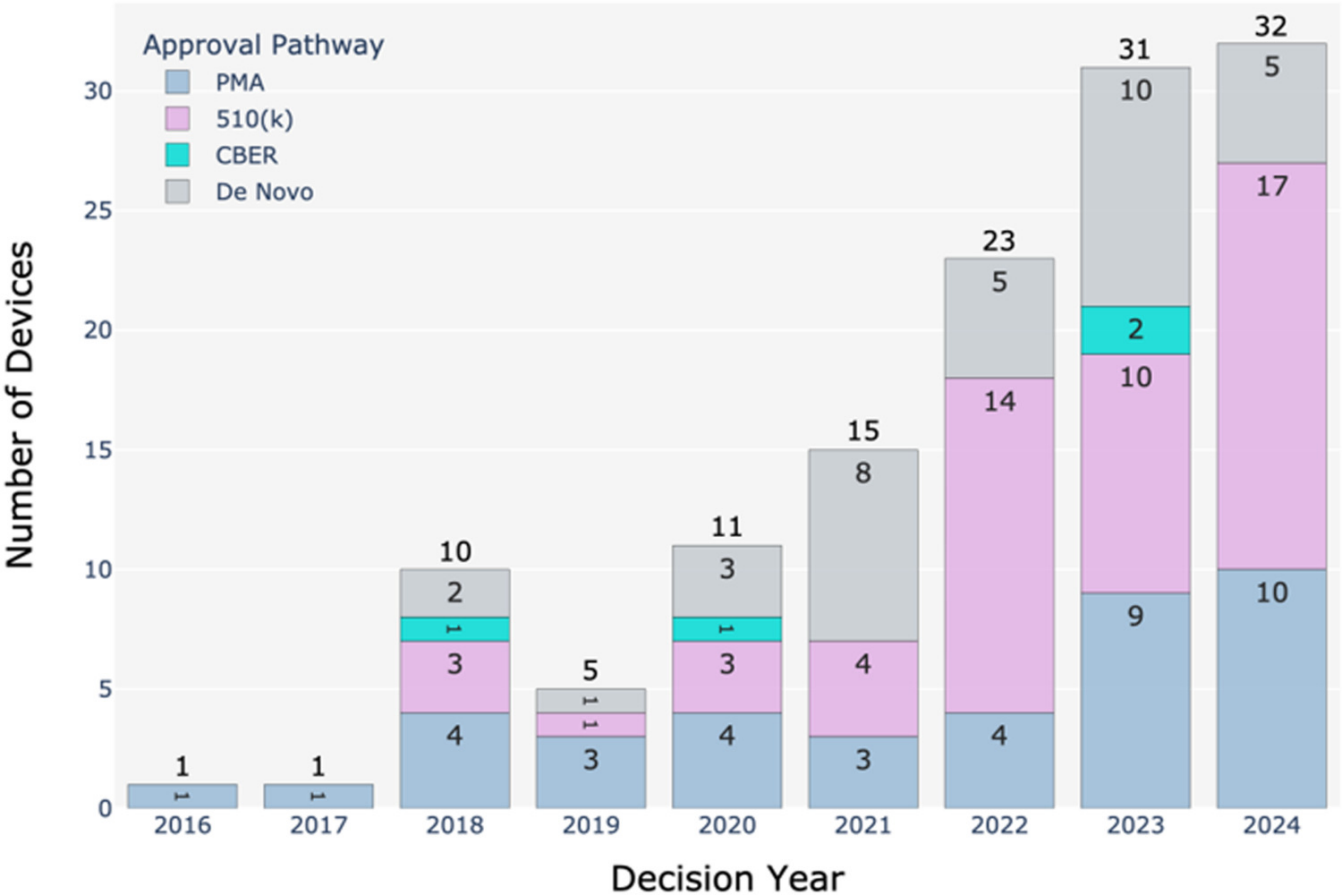
Number of BDP devices in the US market from 2016 to 2024 approved through different FDA regulatory pathways. Data sourced from the publicly available FDA's Breakthrough Devices Program website (1).

Figure 2 illustrates the number of BDP devices in the US market approved through different FDA pathways from 2016 to 2024. The total number of devices receiving marketing authorization has increased significantly, from just one device each in 2016 and 2017 to 32 devices in 2024. This growth shows the role of BDP in accelerating the approval of innovative medical devices. The average number of PMA approvals was around 4 per year from 2018 to 2022, but this number increased to 9 in 2023 and 10 in 2024. This suggests a rising demand for high-risk device approvals. The approvals through the 510(k) pathway increased substantially from 4 in 2021 to 17 in 2024. This pathway is preferred for low to moderate risk devices with suitable predicate. *de novo* approvals have ranged from 5 to 10 in recent years. This pathway is used for novel devices without predicates. The data shows that the 510(k) pathway remains the most frequently used, followed by PMA and then *de novo*. Of the 128 marketed BDP devices, 41% used the 510(k) pathway. This aligns with historical trends where 510(k) submissions are more common due to being faster and less expensive than PMA (6).

The mean decision times for BDP-approved 510(k), *de novo*, and PMA requests from 2018 to 2024 are 152, 262, and 230 days, respectively (1). For comparison, the mean review times for non-BDP devices are 150, 338, and 399 days for 510(k), *de novo*, and PMA (6). Thus, it is evident that the FDA prioritizes the review of submissions for breakthrough devices over others, especially for *de novo* and PMA submissions. However, the reason for similar review times for 510(k) submissions between BDP and non-BDP devices could be due to the inherent simple and streamlined nature of the 510(k) pathway that may leave less room for acceleration under the BDP framework. This highlights how the FDA's prioritization of breakthrough status is particularly impactful for more complex submission types that require rigorous evaluation.

The safety and post-market performance of BDP devices are also critically evaluated, as the expedited nature of their review may raise concerns about long-term safety and effectiveness. To date, eight BDP devices have been recalled, indicating that even with prioritized review, robust post-market monitoring is essential, particularly for innovative medical technologies. Notably, four of these recalls were approved via the 510(k) pathway, while four were approved through the more stringent PMA pathway (1). This distribution suggests that safety concerns can also arise in high-risk devices that have undergone extensive premarket evaluation. The FDA's Manufacturer and User Facility Device Experience (MAUDE) database provides ongoing tracking of device reports of adverse events, enabling the timely identification of potential safety concerns (7). A study by Johnston et al. (2020) highlighted that while the BDP expedites market entry, it also shifts a significant portion of safety evaluation to the post-market phase (8). The relatively low number of recalls suggests that the program's rigorous initial evaluation maintains a strong safety profile, but continued monitoring remains crucial for emerging medical technologies.

**Figure 3:**
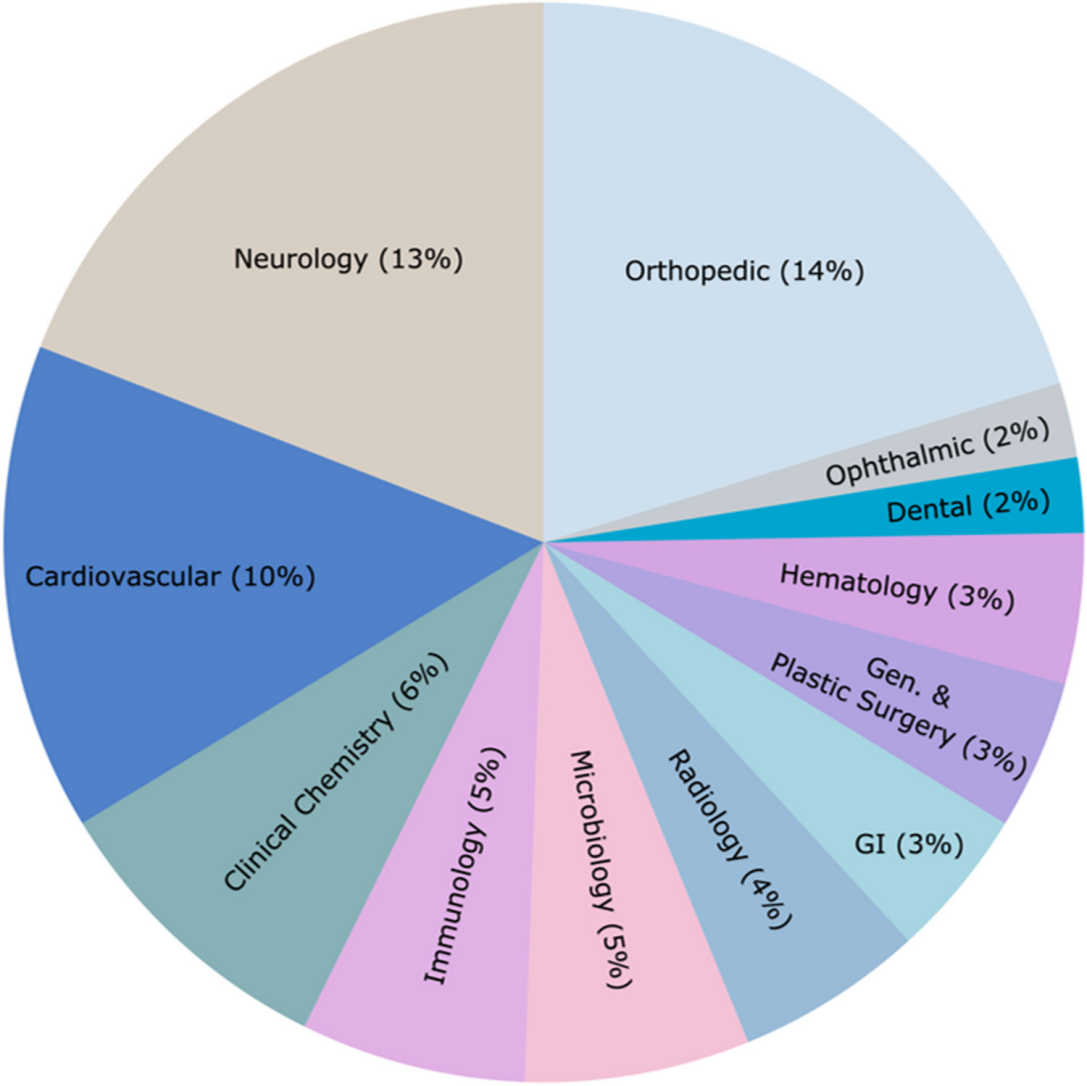
Distribution of market-authorized BDP-designated devices in the US from 2016 to 2024 across various clinical areas. Data sourced from the publicly available FDA's Breakthrough Devices Program website (1). Of the 128 total authorized devices, clinical areas with only one device each and the “Other” category (*n* = 32) are excluded for clarity.

The distribution of BDP marketing authorizations across various clinical areas, as shown in Figure 3, provides an overview of the device categories that are more frequently submitted and approved. The leading clinical areas receiving marketing authorization since the initiation of BDP are orthopedic (*n* = 18), neurology (*n* = 17), and cardiovascular (*n* = 13) devices, collectively accounting for nearly 38% of all authorized devices. Other significant areas include immunology, microbiology, and radiology, reflecting the diverse unmet medical needs being addressed. Clinical categories such as pathology, toxicology, physical medicine, obstetrics/gynecology, ear, nose and throat, clinical toxicology, and anaesthesiology had only one device each and are excluded from the figure. Additionally, 32 devices belonging to a range of uncategorized clinical areas are grouped under the “Other” category, which is not displayed in the figure. This distribution highlights certain clinical areas with high unmet needs being prioritized. At the same time, there is a relative scarcity of approved innovative devices in other specialties, emphasizing opportunities for innovation in these areas.

### 3.1 Enhanced review process under BDP

The Breakthrough Devices Program enhances the FDA's review process by several mechanisms.

#### 3.1.1 Interactive communications

FDA is involved in interactive and timely communication with sponsors throughout all stages of device development and regulatory submission. To facilitate effective collaboration, the FDA and sponsors agree on interaction goals, establish feasible response timelines, and utilize tools like summary tables and “track changes” to streamline discussions. The FDA assigns specialized review teams with relevant expertise and experience to address questions by institutional review committee and may consult external experts for devices with novel scientific aspects. Sponsors are notified in advance of these consultations, given the opportunity to recommend experts, and are provided with details of any decisions influenced by such consultations, ensuring transparency and collaboration throughout the review process (9).

To support sponsors needing timely resolution of potentially novel issues, the FDA also offers “sprint discussions” aimed at reaching mutual agreement on a specific topic within a set time. These discussions are highly interactive, allowing sponsors to provide additional information or revisions to initial proposals, and are designed to address a single topic with specific goals, such as animal study protocol design.

Senior management is involved in the review process to ensure consistent application of program principles and to facilitate efficient development of the device. They also get involved for timely dispute resolution. This support enhances the FDA's ability to address complex challenges associated with breakthrough technologies (9).

#### 3.1.2 Premarket and postmarket balance

For Breakthrough Devices subject to PMA, the FDA continues to require reasonable assurance of safety and effectiveness at the time of approval. When scientifically appropriate, the FDA may rely on postmarket data collection to address uncertainties with pre-market data, allowing earlier access to devices that address unmet medical needs. This approach is a part of the FDA's benefit-risk determination and facilitates faster availability of life-saving devices (9).

#### 3.1.3 Flexibility in clinical study design

The BDP allows for flexibility in clinical study design, which can be particularly beneficial for innovative devices that may not fit traditional study protocols. Manufacturers can discuss and agree on alternative study designs with the FDA, enabling them to generate the necessary clinical evidence more efficiently. This approach includes using prespecified endpoints for clinically meaningful effects, intermediate or surrogate endpoints likely to predict clinical benefit, composite endpoints with justified effect sizes, and adaptive study designs. Such flexibility helps streamline clinical trials without compromising the quality of evidence needed for regulatory decisions (9).

#### 3.1.4 Priority review

The FDA also ensures prompt decision-making on breakthrough device designation requests, with a statutory requirement to render a decision within 60 days of receipt (5). Devices designated as Breakthrough Devices receive priority review, placing their submissions at the top of the review queue and allocating additional resources to expedite evaluation. While priority review aims to accelerate patient access, the review may take longer than standard devices due to the novel scientific and regulatory challenges these technologies often present. However, early and frequent interactions between the FDA and sponsors during development can help streamline the process and ensure timely delivery of safe and effective innovations to patients (9).

#### 3.1.5 Manufacturing and quality system compliance

FDA emphasizes expedited review of manufacturing and quality systems compliance for breakthrough devices, balancing rigorous standards with flexibility to facilitate timely patient access. Sponsors must conform to Quality System regulations (QS Reg) and provide essential manufacturing information, though alternative approaches may be accepted. For instance, if a manufacturer has a good track record for quality system compliance or if the manufacturing sites were recently inspected with favorable outcomes, the FDA may conduct post-approval inspections within 12 months. However, for sites without a recent inspection history or with compliance issues, inspections are typically required pre-approval (9). This reduced burden can accelerate the approval process without compromising safety and efficacy standards.

### 3.2 Funding and coverage

To promote quicker clinical adoption and access to the most advanced devices, the US Centers for Medicare and Medicaid Services (CMS) has implemented several initiatives aimed at facilitating coverage and reimbursement for breakthrough devices. The pathway from FDA approval to Medicare coverage and reimbursement has been complex and evolving.

In 2020, CMS waived the requirement that breakthrough devices demonstrate “substantial clinical improvement” to qualify for additional Medicare reimbursement. This policy change allows market authorized breakthrough devices to be eligible for the New Technology Add-on Payment (NTAP). Hospitals receive supplementary payments and are encouraged to integrate these innovative devices into clinical practice, thus allowing Medicare beneficiaries faster access to breakthrough technologies. The waiver is applicable for a period of up to two years following market authorization (10).

The Medicare Coverage of Innovative Technology and Definition of “Reasonable and Necessary” (MCIT/R&N) rule was published in January 2021 to provide immediate national Medicare coverage for breakthrough devices for up to four years upon FDA market authorization. However, it was repealed before it took effect, due to concerns about the lack of information specific to the Medicare population and potential unknown and unexpected risks to patients (11).

In response to the repeal of MCIT, CMS is exploring alternative coverage mechanisms, including the Transitional Coverage for Emerging Technologies (TCET) pathway. It was proposed in June 2023 and finalized in August 2024, marking a step towards addressing the coverage gap for breakthrough devices. This initiative aims to provide a more balanced approach to coverage for certain breakthrough devices. This voluntary program utilizes existing National Coverage Determination (NCD) and Coverage with Evidence Development (CED) processes to streamline coverage decisions. TCET would enable CMS to support limited coverage for new technologies while additional data on safety, effectiveness, and clinical utility is gathered, ensuring that breakthrough devices with promising clinical benefits are made available to patients more quickly. By offering temporary coverage, TCET could encourage broader clinical adoption and enhance access to innovative devices until full Medicare coverage is warranted. The pathway offers manufacturers increased opportunities for pre-market engagement with CMS and provides flexibility in addressing evidence gaps to support Medicare coverage. To manage resources effectively, CMS anticipates accepting up to five TCET candidates annually, with the goal of finalizing NCDs within six months of FDA market authorization for technologies accepted into and continuing in the TCET pathway (12, 13).

The Ensuring Patient Access to Critical Breakthrough Products Act, introduced in Congress, aims to provide prompt Medicare coverage for breakthrough devices for four years following FDA approval (14). If passed, this legislation could significantly impact the coverage landscape for breakthrough devices.

### 3.3 Criticisms, ongoing efforts, and proposed reforms

The BDP has been praised for expediting patient access to innovative medical technologies. However, it has drawn criticism for shortcomings in safety, effectiveness, transparency, and equitable access, prompting calls for reform.

There is limited public access to details about devices receiving breakthrough designation, their postmarket performance, or confirmatory trials. While the FDA collects this data to ensure ongoing safety and efficacy, it is not made publicly available, which is often seen as a lack of transparency. Many breakthrough devices enter the market with limited premarket evidence, including randomized controlled trials (RCTs), or rely on surrogate endpoints, leading to concerns about safety and reliability of clinical outcomes (8). This causes a shift in the burden of proving safety and efficacy from the premarket phase to the postmarket phase, where enforcement is less stringent. Postmarket surveillance systems may not adequately capture adverse events or measure device performance across diverse populations. Moreover, the FDA and manufacturers face challenges in promptly identifying affected patients and addressing recalls effectively. This raises concerns about device safety and effectiveness and disparities for underserved populations (15).

There are concerns about the economic and equity implications of the BDP due to limited clinical evidence used to approve these devices and its financial impact on public healthcare systems A recent study states that many cardiovascular devices designated as breakthrough have been approved based on single-arm trials that focus on surrogate endpoints and short-term follow-ups, with trial participants often unrepresentative of Medicare beneficiaries (16). This has allowed manufacturers to set high device prices while also qualifying for automatic Medicare reimbursement, resulting in increased short-term and long-term costs for CMS and health systems (16). Critics argue that these practices put Medicare beneficiaries at risk by exposing them to devices with unverified benefits. This also aggravates equity concerns, as certain populations may be disproportionately affected.

While these critiques highlight significant concerns about the BDP, the FDA recognizes the potential risks associated with accelerated approvals. The FDA's ongoing efforts demonstrate a commitment to balance expediting innovative medical technologies and ensuring patient safety. The agency requires manufacturers to develop comprehensive post-market surveillance plans that include:

• Reporting Requirements: Breakthrough devices are tracked through the MAUDE database for adverse event reporting.
• The FDA maintains the authority to require additional clinical data, modify device labelling, issue safety communications and initiate device recalls.

The FDA's commitment to post-market surveillance is evident in its ongoing monitoring of the 128 marketed breakthrough devices, with eight devices recalled to date. Moreover, the agency continues to refine its approach. The September 2023 guidance update explicitly emphasized considerations for health equity and diverse patient populations, demonstrating an evolving approach to device evaluation.

The concerns emphasize the need for reforms such as making the breakthrough designations public, mandatory postmarket evidence generation and its public disclosure, diversifying clinical trials, mandatory federal rebates until completion of confirmatory clinical trials or until certain postmarket requirements are met, outcome-based reimbursement models, and penalizing delayed confirmatory studies. These measures will incentivize evidence generation, and ensure patient safety, transparency, and equitable allocation of healthcare resources.

## 4 Accelerated approval in the European Union (EU)

Although there are no specific accelerated approval pathways for medical devices at the EU level, several initiatives have been implemented to ensure timely and coordinated access to innovative medical devices while maintaining rigorous standards for safety and efficacy. The European Union Medical Device Regulation (EU MDR) and the Health Technology Assessment Regulation (HTAR) play pivotal roles in streamlining the pathway from device development to patient access. Central to this process is the CE (Conformité Européenne) mark, which manufacturers can affix to their medical devices once they have successfully passed a conformity assessment. This assessment typically involves an audit of the manufacturer's quality management system and a review of technical documentation on the device's safety and performance (17). Accredited notified bodies (NBs), designated by EU Member States, guide companies and conduct these conformity assessments (18).

EU MDR came into force in May 2021, replacing the previous Medical Device Directive (MDD). It introduced stricter requirements for the certification of medical devices to enhance patient safety while fostering innovation. Although this ensures high safety and performance standards, NBs have been criticized for delayed patient access due to resource constraints and their limited number (19). To address the bottlenecks, the European Commission has been working to streamline review processes, increase the number of certified NBs, expand their capacity, and promote better coordination between them. The number of NBs designated under MDR has increased from about 20 in 2021 to 50 in 2024 (18). This expansion aims to enhance the capacity for conformity assessments and timely availability of medical devices in the EU market.

To further expedite access to innovative medical devices, the EU has introduced the HTAR, which entered into force in January 2022. Its phased implementation began in January 2025, with coordinated evaluation of high-risk medical devices, drugs, and *in vitro* diagnostic devices across EU member states (2). The most significant impacts of HTAR are the introduction of joint clinical assessments (JCA) and joint scientific consultations (JSC).

The JCAs will be conducted at the centralised EU level and shared among member states, reducing the duplication of national evaluations (20). The European Commission has defined timelines and steps for conducting JCAs, which will provide scientific evidence to member states' authorities at an early stage after a device's marketing authorization (21). JCAs will initially focus on certain medicinal products in 2025 and will expand to include selected medical devices from 2026 (22). For medical devices, the European Commission has specified that JCAs will apply to certain high-risk devices that have undergone expert panel consultation during their conformity assessment (2, 20). The criteria for devices to be included in the JCA are being developed based on clinical relevance, degree of innovation, and potential for cross-border healthcare impact. JCAs will focus on the assessment of the relative clinical effectiveness and safety of the new health technology compared to existing alternatives. This process involves a single dossier submission by manufacturers and centralized review by designated EU HTA bodies, according to strict timelines defined by the European Commission (21, 22). By pooling clinical evidence and expertise, JCAs aim to deliver high-quality, consistent assessments that inform national reimbursement decisions, thus reducing repetitive national evaluations. This coordination can help reduce delays in national assessments, support faster market access, and promote EU-wide efficiency.

Manufacturers of devices that meet certain criteria (unmet medical needs, first in class, major impact on patients, public health or healthcare systems, etc.) can request JSCs, which enable early discussions with HTAR and clinical experts. Manufacturers can seek JSCs that are designed to help optimize evidence generation plans before pivotal clinical investigations, thus enhancing the quality of evidence preparation for subsequent JCA (23, 24). Through JSCs, manufacturers can consult with representatives from the Coordination Group on Health Technology Assessment (HTACG) and clinical experts to discuss pivotal clinical investigation plans, comparators, endpoints, and study populations before starting their main studies (24). JSCs can take place in parallel with the consultation of an expert panel. While non-binding, these consultations provide valuable direction for optimizing clinical development strategies. Early engagement through JSCs can help align clinical study design aspects or clinical investigation design aspects with both regulatory and HTA evidence requirements, potentially reducing the need for additional studies in the assessment phase and accelerating the path to market access (23, 24).

The effective implementation of HTAR will require significant collaboration between EU member states, regulatory bodies, and manufacturers. While the HTAR aims to streamline the assessment process and reduce duplication of efforts, it is important to note that member states retain the authority to make non-clinical assessments (such as social implications, economic, and ethical aspects) and make final decisions on pricing, reimbursement, and coverage (19). The member states may request additional data for these assessments. This can lead to significant disparity across European countries and cause delays in public access.

It is important to note that there exists an accelerated assessment scheme by European Medicines Agency (EMA) called PRIME (PRIority MEdicines), which primarily focuses on medicinal products. However, it is relevant for certain medical devices that are integrated with medicinal substance (combination products) that target an unmet medical need (25). These combination products could potentially benefit from the enhanced support and early dialogue with regulators that PRIME offers. Thus, manufacturers of eligible combination products may consider PRIME as part of their regulatory strategy to accelerate the path to market.

The EU's efforts to harmonize health technology assessment reflect a balance between centralized processes and national healthcare decision-making. While this approach respects the diverse healthcare systems across member states, it also presents challenges for achieving accelerated access to innovative medical devices throughout the EU. To address these challenges, the adoption of a more centralized EU pathway specifically designed for innovative devices is recommended. This would eliminate the current disparities between member states and provide manufacturers with a clear and consistent route to market. Moreover, the enforcement of uniform standards across the EU would also enhance patient safety and facilitate more efficient post-market surveillance and data collection.

An important step towards this is European Database on Medical Devices (EUDAMED), a comprehensive IT system established by the EU MDR. EUDAMED has six interconnected modules covering various aspects of the medical device lifecycle, from registration to market surveillance (26). While some modules have been available for voluntary use since 2020, the full implementation of EUDAMED, including the remaining modules for Vigilance, Clinical Investigation & Performance Studies, and Market Surveillance, is expected to become mandatory around 2027. Once fully operational, EUDAMED will serve as a centralized hub for information on medical devices, manufacturers, certifications, and post-market activities. This database will enhance transparency and improve patient safety and traceability of medical devices across the EU.

### 4.1 Funding and coverage

Some EU countries provide early coverage schemes for promising medical technologies and procedures. It is essential to note that these programs aim to expedite financial access, but they do not necessarily accelerate the regulatory approval process itself or guarantee timely access.

#### 4.1.1 France

In France, all HTA activities are managed by the French National Health Authority (Haute Autorité de Santé or HAS), which was established in 2004. France has implemented several innovative funding and coverage initiatives to accelerate access to medical devices, including Prise en Charge Transitoire (PECT), Innovation Funding (IF), and Prise en charge anticipée numérique (PECAN).

##### 4.1.1.1 Prise en Charge Transitoire (PECT)

The Transitional Coverage (Prise en Charge Transitoire or PECT) scheme, established in 2021, provides temporary reimbursement for CE-marked medical devices for up to 12 months before potential permanent inclusion in the List of Reimbursable Products and Services (LPPR) (27). The eligibility criteria for transitional coverage under PECT focus on addressing serious or rare diseases or compensating for disabilities, targeting unmet or poorly met medical needs. Eligible technologies must demonstrate potential for significant improvement in health outcomes or disability compensation and exhibit innovation beyond mere technical advancements. Clinical studies should provide evidence of clinically relevant efficacy and acceptable safety profiles, with ongoing research expected to generate sufficient data for evaluation within 12 months. Digital medical devices with therapeutic purposes or those used for remote medical monitoring are excluded from eligibility (28).

The application must be submitted through the online platform Sésame. Simultaneously, an application must also be submitted to the Digital Health Agency (ANS – Agence du numérique en santé) to assess compliance with security and interoperability standards. Manufacturers can request a free and confidential meeting with HAS before submission of the application. An Early dialogue meeting can be requested for assistance regarding study protocol, while a Pre-submission appointment assists with information on preparing the file (28). The eligibility criteria are assessed within 60 days by the Medical Device and Health Technology Evaluation Committee (CNEDiMTS), a HAS committee, for reimbursement by national health insurance (29). The reimbursement includes the negotiated price of the medical device agreed upon by the Ministry of Health and the manufacturer. The broader healthcare costs associated with the device are not covered (30). Between 2021 and 2023, 6 devices were granted a positive evaluation (31).

##### 4.1.1.2 Innovation Funding (IF)

IF, established in 2009, provides temporary coverage for the duration of the clinical trial to innovative medical devices, *in vitro* devices, and medical procedures during the data-gathering phase. Some of the eligibility criteria for a device to be considered innovative include demonstration of the innovative nature of the technology, comprehensive assessment of patient safety and device risks, and potential ability to fulfill unmet medical needs or reduce healthcare expenditures. HAS and the Ministry of Health assess compliance with these criteria. The coverage is for technologies that are in the early stages and lack sufficient data for full reimbursement. It is a coverage with evidence development (CED) scheme, where companies commit to conducting trials to gather additional clinical or medico-economics evidence (32). For the medico-economic (cost-effectiveness) evidence, the new technology must be at least as effective in clinical terms as the existing standards of care (33). Thus, IF facilitates French patients' early access to promising technologies and assists authorities in making informed decisions regarding mainstream funding (i.e., inclusion in the LPPR) based on collected data (30).

Similar to PECT, companies can request assistance from HAS prior to submission of an application for funding. Assistance is possible in the form of two types of meetings: (1) Pre-submission appointments – for information on technico-regulatory aspects and (2) Early dialogues – for matters concerning the clinical development of the technology or about the conduct of medico-economic study. After application, HAS issues a favorable or an unfavorable opinion based on eligibility criteria within 75 days, following which the Ministry of Health conducts budget analysis and makes a decision on granting funding in 75 days (32). The IF provision includes either full or partial coverage of the medical device (MD) and associated patient care (only French patients are covered) during the study, as well as for an additional cohort to ensure uninterrupted access until the device receives reimbursement (30). As of 2023, HAS has granted a favorable opinion to 34 health technologies (including 21 devices and 13 medical procedures) (34).

##### 4.1.1.3 Prise en charge anticipée numérique (PECAN)

PECAN, introduced in 2022, allows for early coverage of CE-certified innovative digital medical devices (DMDs) for one year before they are permanently reimbursed under the French healthcare system. The program covers digital health applications (intended for inclusion in the LPPR) and remote health monitoring systems (intended for List of Medical Telemonitoring Activities or Liste des Activités de Télésurveillance Médicale, LATM). Parallel assessments are performed by CNEDiMTS for eligibility and Digital Health Agency (ANS) for security and interoperability (30). The innovative nature is evaluated based on available data from ongoing studies and taking into account possible relevant comparable product, procedure, or service (35). Following the opinion by CNEDiMTS, the Ministers of Health and Social Security confirm eligibility for granting the non-extendable reimbursement. Following the PECAN reimbursement, the applicant must deliver evidence of clinical benefit and submit LPPR inclusion request within 6 months or LATM inclusion request within 9 months. The requirements for study design are flexible with multicentric RCTs, although still considered as the highest level of evidence, are not mandatory (36).

In April 2024, the reimbursement to the manufacturer or retail distributor of therapeutic digital medical devices under PECAN was announced. The compensation includes an initial amount of €435 including tax, once per patient, for a maximum of 3 months, followed by €38.3 monthly including tax, resulting in a maximum annual compensation of €780 per patient. The compensation by national health insurance for early coverage is subject to the actual use of the device (37). The reimbursement system for remote medical telemonitoring under PECAN is structured with a progressive scale where providers receive a monthly flat rate of €50 per patient for up to 4,999 patients. The rates are based on the average monthly active queue over a six-month reference period. As the number of active patients grows, the compensation per patient decreases (38).

#### 4.1.2 Germany

The 2019 Digital Healthcare Act (Digitale-Versorgung-Gesetz, DVG) introduced the Digital Health Applications (Digitale Gesundheitsanwendungen, DiGA) program in Germany which came into force in 2020, establishing a model for integrating digital therapeutics into the healthcare system (39). This innovative approach was the first in the EU countries to allow accelerated approval and reimbursement of certain digital health applications. The DiGA Fast Track process for DMDs, overseen by the Federal Institute for Drugs and Medical Devices (Bundesinstitut für Arzneimittel und Medizinprodukte, BfArM) (40), enables rapid assessment (within three months) and provisional reimbursement for one year (with a possible 12-month extension) before the full data is collected to assess long-term value. Devices or applications that qualify for DiGA reimbursement can be included in the DiGA directory (a national directory), provided they meet certain requirements. It must be a medical device of risk class I or IIa under the MDR, primarily based on digital technologies, and intended for recognizing, monitoring, or treating diseases or disabilities (41). The DMD must demonstrate positive healthcare effects (positive Versorgungseffekte, pVE), either through medical benefits or improvements in structure and processes between patients and healthcare providers (patientenrelevante Struktur- und Verfahrensverbesserungen, pSVV). Evidence of positive healthcare effects must be provided through clinical trials or studies, with temporary inclusion in the DiGA directory allowed based on preliminary evidence, but for permanent inclusion, high-quality evidence must be submitted within 12 months. Additionally, it must comply with data protection, security, and interoperability standards (41).

The program assists developers of digital health applications. The Innovation Office provides guidance in the form of kick-off meetings for early-stage regulatory orientation and DiGA-specific consultations addressing technical, evidence-related, and procedural queries (42). This accelerates the development of compliant and innovative digital health solutions. Once listed in the DiGA directory, physicians and psychotherapists can prescribe these digital health applications, and health insurance covers the costs (43).

Initially, manufacturers set the prices for DiGAs for twelve months. Subsequently, the reimbursement is negotiated between the manufacturers and the National Association of Statutory Health Insurance Funds (GKV-SV) (41). From 2026, at least 20% of a DiGA's reimbursement will be performance-based, aiming to align payment with effectiveness. Additionally, recent legislation has introduced maximum price reimbursement thresholds for categories that include at least two DiGAs. The system also allows for special considerations in pricing for DiGAs that are first in their indication group, target rare diseases, or utilize advanced artificial intelligence to incentivize innovation in these areas (39). This evolving landscape for DiGAs in Germany shows ongoing efforts to balance innovation, clinical effectiveness, and cost-efficiency within the healthcare system. A new Digital Health Act (Digital-Gesetz, DigiG) came into effect in March 2024 to further accelerate the digitalization of the healthcare system. Key components of the Act are the establishment of electronic patient record (elektronische Patientenakte, ePA) for all insured patients, beginning in 2025, and the setting up of e-prescription as a binding standard (44). It will facilitate the sharing of health data and offer targeted support for patient care.

#### 4.1.3 Belgium

Belgium has implemented several coverage schemes to facilitate accelerated access to innovative medical devices. The National Institute for Health and Disability Insurance (NIHDI) plays a key role in evaluating and approving medical technologies that can be added to the list of reimbursable devices to be covered by national health insurance. One such scheme is the Restricted Clinical Application (Application Clinique Limitée) for invasive medical devices and implants. This program provides temporary reimbursement for promising technologies to generate additional evidence in a limited number of hospitals for 3–5 years when there is uncertainty about their added value compared to existing technologies. After the completion of coverage period, a decision is made about permanent reimbursement (45). Belgium has recently launched initiatives to streamline the integration of digital health technologies into the Belgian healthcare system.

As of October 2023, manufacturers, scientific associations, professional organizations, and hospitals can apply directly to the NIHDI for reimbursing CE-marked medical mobile applications (46). This scheme aims to address the challenge of evaluating and reimbursing digital health solutions. The mHealth pyramid is an initiative to centralize information on mobile health apps and provide a validation pyramid for digital health technologies, facilitating their integration into the Belgian healthcare system. The pyramid consists of three different certification levels with increasingly stringent requirements (36). The application for provisional or permanent reimbursement is submitted to NIHDI and evaluated by a multidisciplinary committee.

#### 4.1.4 Austria

Austria has implemented a limited approach to innovative payment schemes for medical devices. The country utilizes a Neue Untersuchungs- und Behandlungsmethoden (NUB) system which aims to provisionally include innovative medical procedures and associated technologies in the basic insurance package while clinical evidence is still being gathered (45). Although the costs of the procedure are covered, NUB does not provide additional funding to incentivize the use of new procedures, and no specific clinical studies are mandated. It is important to note that the NUB covers medical technologies in the context of their use within new diagnostic or therapeutic methods, rather than as standalone devices.

In the inpatient sector, reimbursement is primarily managed through diagnosis-related group (DRG) adjusted budgets, with some highly innovative devices being individually reimbursed through the Medizinische Einzelleistungen (MEL) catalogue (47).

#### 4.1.5 Netherlands

The National Healthcare Institute (Zorginstituut Nederland, ZIN) plays a crucial role in recommending coverage for innovative devices in the Basic Health Insurance package and encouraging the use of digital care (48). The Netherlands has a “Small-Scale Experiments for the Introduction of Innovations” framework managed by the Dutch Healthcare Authority (NZa) that allows healthcare providers and insurance companies to conduct short-term, small-scale projects to test and introduce new healthcare practices or technologies that might not have enough evidence to be covered under standard health insurance. The project is covered for a maximum of three years with the potential of extension up to 5 years (49).

As of September 2024, the Dutch Ministry of Health, Welfare and Sport (VWS) decided to discontinue the “Promising Care” subsidy scheme, which aimed to accelerate patient access to innovative medical technologies by funding clinical studies and associated patient care. The ZIN is currently in discussions with VWS to explore alternative public funding mechanisms to continue supporting research of promising healthcare interventions (50).

These coverage schemes demonstrate efforts by several EU member states to facilitate early patient access to innovative medical technologies while ensuring robust evaluation of their clinical and economic impact, although implementation varies significantly across the EU.

## 5 Risks and challenges associated with accelerated pathways

Accelerated pathways for medical devices, including the FDA's Breakthrough Devices Program and similar programs in other regions, expedite access to innovative technologies but also introduce significant regulatory risks and post-market surveillance challenges. One major concern is the reliance on limited clinical data or surrogate endpoints. Devices approved through these pathways often use short-term indicators or smaller studies to predict effectiveness (8). This may lead to unforeseen safety issues or limited efficacy in broader patient populations post-market. For instance, a study revealed that shorter FDA review times for high-risk cardiovascular devices are associated with a higher likelihood of adverse events (51). In global context, this can have a disproportionate impact on vulnerable regions and populations where access to healthcare and regulations may vary significantly.

As the pre-market studies of devices approved through accelerated pathways are less comprehensive, robust post-market surveillance studies become necessary. However, global regulatory agencies face significant challenges in monitoring the real-world performance of these medical devices due to resource constraints. For example, the EU's EUDAMED database, which is being designed to enhance coordination regarding medical devices with a focus on patient safety and clinical evidence, has faced implementation delays. Full implementation of EUDAMED, including its crucial market surveillance module has faced technical challenges associated with developing a system capable of handling large volumes of market surveillance data across EU member states leading to extended transition periods. The initial expected year for EUDAMED's full functionality was 2024 which has now been postponed to at least 2027 (52). The phased development and implementation of EUDAMED illustrate the challenges in establishing comprehensive monitoring systems.

Thus, there is a need for a balanced approach to innovation and safety. These pathways can benefit from international collaboration on data sharing, especially for rare conditions with limited data.

## 6 Organizational implications of accelerated pathways

The regulatory landscape for medical devices has significant implications for organizational structure, resource allocation, and strategic planning across different types of device developers. While accelerated pathways offer opportunities for faster market entry, they also introduce complex organizational challenges for manufacturers and public research institutions.

### 6.1 Manufacturers

Medical device companies must strategically align their development processes with the requirements of programs like the FDA's Breakthrough Devices Program or the EU's evolving accelerated access initiatives. These pathways often demand early and frequent engagement with regulators (9), robust project management, and the ability to generate and manage clinical evidence within strict timelines (4). Companies need to allocate significant resources to ensure the timely completion of confirmatory trials in the US or to prepare for joint clinical assessments (JCA) and expert panel reviews in the EU (19, 20). This can mean increased investment in regulatory affairs team, technology for data collection and real-world evidence, partnerships with contract research organizations, and reallocating resources from established product lines to breakthrough innovations. The resource burden may be particularly acute for small and medium-sized enterprises (SMEs), potentially requiring difficult trade-offs between product development and regulatory compliance, or strategic collaborations to share compliance costs (53).

Accelerated pathways also influence business strategy. In the US, the centralization of FDA's BDP allows for more predictable planning, but companies must be prepared for rigorous compliance standards, post-market obligations and the risk of market withdrawal if confirmatory evidence is lacking. In the EU, the decentralized system and evolving HTA requirements make it complicated for manufacturers to navigate varying national expectations, affecting market access strategies (53). Manufacturers need to address both regulatory and reimbursement evidence requirements, which require integration of clinical, regulatory, and health economics expertise.

### 6.2 Public research institutions

Academic and public research innovators face distinct challenges. While they are often strong in technical innovation, they lack the dedicated regulatory infrastructure of commercial manufacturers. They may benefit from early engagement with regulators but may lack institutional frameworks to support this effectively. Moreover, the need for comprehensive clinical data, and post-market surveillance can strain academic resources and may necessitate partnerships with industry. Also, research timelines and funding may not align with regulatory submission timelines.

Ultimately, the successful implementation of accelerated approval strategies requires organizations to be well-resourced and strategically aligned across regulatory, clinical, and commercial functions. Also, it is beneficial for organizations to engage early not only with regulatory authorities but also with health insurers and national health systems to understand their evidence expectations.

## 7 Global convergence and recommendations

Global convergence in medical device regulation is increasingly recognized as a crucial step towards accelerating access to innovative medical technologies globally. There is an urgent need for regulatory harmonization to streamline approval processes and ensure patient safety across nations. The primary goal is aligning regulatory requirements and standards for medical devices across different countries, reducing redundancies, and expediting market access for safe and effective medical devices (54). This approach can significantly decrease the cost of regulatory compliance for manufacturers while allowing earlier access to new technologies and treatments.

The development and adoption of internationally harmonized regulations and criteria for medical device assessment can reduce regulatory barriers and facilitate global market access. This approach will also ensure consistent safety and efficacy standards across borders. A key strategy for optimizing resource utilization and accelerating approval processes is encouraging regulatory authorities to consider assessments and decisions made by trusted agencies of other countries, a practice known as regulatory reliance (55). This will particularly benefit countries with limited regulatory resources in making informed decisions about medical device approvals (56). Moreover, establishing Mutual Recognition Agreements (MRAs) between nations could further streamline market entry reducing duplication of efforts, for instance, redundant evaluations (57). Accelerating collaborative dynamics such as knowledge exchange and expertise sharing through regular scientific discussions among global regulatory authorities and industry stakeholders can foster a harmonized regulatory environment for expedited development and commercialization plans. This collaboration can lead to the development of best practices, shared standards, and more efficient regulatory processes. It could also potentially lead to the development of protocols for multiregional clinical trials which could expedite access worldwide.

Initiatives like the International Medical Device Regulators Forum (IMDRF) are working towards harmonizing regulatory requirements across different jurisdictions including Australia, Brazil, Canada, China, Europe, Japan, Russia, Singapore, and the United States. IMDRF-developed programs like the Medical Device Single Audit Program (MDSAP) demonstrate the potential of such collaborative efforts in reducing regulatory burden while maintaining high quality and safety standards (55). The MDSAP enables recognized auditing organizations to conduct a single audit of a medical device manufacturer that covers the quality management system requirements of participating regulatory authorities, including those of Australia, Brazil, Canada, Japan, and the United States.

Global convergence must extend beyond market entry and include harmonized approaches to post-market surveillance. This lifecycle approach is crucial for maintaining public trust and ensuring long-term effectiveness of devices worldwide. By implementing globally consistent monitoring systems, regulators and manufacturers can more effectively track device performance and safety across diverse populations and healthcare settings. It will also provide valuable data for continuous improvement of devices and regulatory processes.

By implementing these strategies of harmonization, reliance, and collaboration, the global medical device community can work towards a more unified regulatory landscape with reduced duplication of efforts, and potentially accelerated access to innovative medical technologies worldwide. However, it is important to note that along with convergence, regulatory authorities must still maintain their ability to make independent decisions based on their specific regional needs and healthcare systems.

## 8 Conclusion

This review highlights the complex regulatory landscape of accelerated access pathways for medical devices in the US and EU.

These programs are characterized by frequent consultations between the regulatory authorities and manufacturers from the early stages of device development. The Breakthrough Devices Program (BDP) in the US has shown promise in reducing approval times. In the EU, the implementation of MDR and HTAR represents a significant step towards harmonization, though disparity in coverage and reimbursement policies across member states continue to impact patient access.

While these programs aim to expedite market entry for innovative devices addressing unmet medical needs, they also present challenges in balancing rapid access with safety considerations. Expedited programs require robust post-market surveillance and real-world evidence collection to ensure long-term safety and effectiveness. The global convergence efforts offer promising avenues for streamlining regulatory processes across regions, potentially reducing duplication of efforts and facilitating faster global access to innovative medical devices.

Moving forward, it is crucial for regulatory bodies, industry stakeholders, healthcare systems, health insurance, and reimbursement authorities to collaborate in refining these pathways. This includes developing more standardized approaches to evidence generation, enhancing post-market surveillance capabilities, and addressing funding and coverage challenges. These approaches would ensure that accelerated approval translates into accelerated patient access to safe medical technologies. With rapid advancements in this sector, accelerated pathways will play an increasingly important role in bringing innovative devices to market. However, their success will depend on ongoing evaluation and adaptation to ensure they meet their intended goals without compromising patient safety or healthcare system sustainability.
